# Long term follow-up after sudden withdrawal from a multicentric study of abatacept in juvenile idiopathic arthritis – data from the Portuguese cohort

**DOI:** 10.1186/1546-0096-9-S1-P191

**Published:** 2011-09-14

**Authors:** S Melo Gomes, JA Melo Gomes

**Affiliations:** 1 Department of Pediatrics, Centro Hospitalar Oeste Norte, Portugal; 2 Pediatric Rheumatology Clinic, Instituto Português de Reumatologia, Lisbon, Portugal

## Background

A cohort of patients participating in a multicentric, multinational phase III trial to assess the efficacy and safety of abatacept in juvenile idiopathic arthritis (JIA) were discontinued from the study due to administrative reasons.

## Aim

To assess long term efficacy and monitor clinical follow up after the sudden removal of abatacept.

## Methods

Patients in the open arm of the trial, receiving 10mg/kg monthly infusions of abatacept were followed for 4 years after its suspension. Outcome measures included clinical remission and disease control without biologics.

## Results

Eight patients (7M/1F) with mean age of 16.5 years (range 13-19years) and disease duration of 8,6years (range 1,5-13years) were followed. All patients had polyarticular involvement only partially responsive to methotrexate (extended oligoarticular (OE)-3, polyarticular Rheumatoid factor IgM negative (Poly)-2, systemic onset (SoJIA)-3). Concurrent therapies, maintained after the abatacept trial included NSAIDS, Methotrexate (8/8) and low dose steroids (3/8).

All patients met the ACRpedi30 criteria, 5 of which (OE and Poly) were either in remission (2/8) or had marked improvement (1or 2 active joints). After 12 months of follow-up, all OE patients had inactive disease, as well as one Poly (4/8). Subsequently, 1of the OE and the other Poly flared, with etanercept being introduced at 27 and 29months of follow-up, respectively; 3 patients currently remain in full remission after 4 years (2 OE, 1 poly).

SoJIA patients maintained active disease, requiring biologic therapy (mean 18months).

## Conclusion

Although it’s a small cohort, this study suggests that abatacept may induce long term remission in polyarticular JIA patients, especially EO and Poly subtypes. Similar results were not observed in SoJIA patients.

